# Social Health Insurance for Universal Health Coverage in Low and Middle-Income Countries (LMICs): a retrospective policy analysis of attainments, setbacks and equity implications of Kenya’s social health insurance model

**DOI:** 10.1136/bmjopen-2024-085903

**Published:** 2024-12-11

**Authors:** Susan Nungo, Jonathan Filippon, Giuliano Russo

**Affiliations:** 1 Wolfson Institute of Population Health, Queen Mary University of London, London, UK

**Keywords:** Health Equity, PUBLIC HEALTH, Health policy

## Abstract

**Abstract:**

**Objectives:**

To analyse the potential of the Social Health Insurance (SHI) model to support the achievement of Universal Health Coverage (UHC) in Low and Middle-Income Countries (LMICs) through a policy analysis case study of Kenya’s National Health Insurance Fund (NHIF).

**Design:**

We used an adaptation of the policy triangle framework to perform a retrospective policy analysis of Kenya’s NHIF, drawing from semistructured interviews and analysis of published documents and grey literature.

**Setting:**

We focused on Kenya’s NHIF as a case study.

**Participants:**

We conducted 21 interviews with key stakeholders including policy experts, healthcare providers and formal and informal sector workers. We then triangulated the interview findings with document analysis.

**Results:**

Only 17% of Kenya’s population are currently covered by the SHI as of 2023. Only 27% of the informal economy is covered by the NHIF, implying very low uptake and/or retention rates. We found little stakeholder engagement in the policy implementation process and minimum adoption of expert advice. Our analysis suggest that political affiliations and positions of power heavily influence health financing policies in Kenya. Purchasing and payment of healthcare was found to be riddled with inefficiencies, including slow bureaucratic reimbursement procedures, little expertise by rural hospital clerks, misappropriations and favouritism of specific private healthcare providers. We also found that group-based parallel schemes and penalty payments for defaulted premiums widened the existing inequity gap in healthcare access.

**Conclusion:**

Although the SHI system is perceived to increase coverage and the quality of health services in Kenya, substantial structural and contextual challenges appear to deter its suitability to finance the attainment of Universal Health Coverage. From Kenya’s experience, we identify little informal sector participation, inefficiencies in purchasing and payment of healthcare services, as well lack of political goodwill, as key bottlenecks for the implementation of SHI schemes in LMICs. LMICs adopting SHI need to also implement co-financing arrangements that do not impose on the population to co-finance, strategic purchasing systems, political goodwill and good governance for the SHI systems to be beneficial.

**STRENGTHS AND LIMITATIONS OF THIS STUDY**

- This study offers an original empirical angle to explore the suitability of the social health insurance financing mechanisms for universal health coverage.
- The number of participants interviewed was limited, and a larger sample might provide a more complete representation of Kenya’s health system stakeholders.
- Due to the narrow political window within which the study was carried out, we were unable to include some historical political actors who played a role in the social health insurance policy process.

## Introduction and background

 Health financing is a core component that determines the ability of a health system to deliver the fundamental right to health.^1^ It involves how health resources are mobilised, pooled and used to deliver quality, acceptable and accessible health, and healthcare.^1^ The World Health Report stipulates that health financing systems should be designed to be able to ‘provide all people with access to needed health services (including prevention, promotion, treatment and rehabilitation) of sufficient quality to be effective’ without exposing the users to health-related financial catastrophes.^2^ The reference to ‘all people’ implies universal, and ‘access’ implies coverage, whereas needed health services imply human need, thus the widely used term, universal health coverage (UHC).^3^

There are, however, conceptual limitations that imply that UHC is only a basic package of healthcare services, mostly linked with primary healthcare, and not precisely comprehensive healthcare or ‘Health for All’.^4^ In its strictest theoretical interpretation, it is debatable if UHC can be fully attained in any country whether high, low or middle income.^3^ Despite this limitation, UHC increasingly received backing from the WHO and other international organisations after the turn of the millennia.^4^ Notwithstanding its conceptual challenges, health financing remains a challenge for LMICs, hence deterring the progress towards UHC and consequently leaving populations exposed to poor health status and health-related financial hardships.^2^ Financing challenges are especially driven by resource shortages, challenges with technology and infrastructure, corruption and inefficiency in the health systems operations in low and middle-income countries (LMICs).^5^ LMICs, by the World Bank definitions, are countries that have a gross national income per capita of below $4515 as of 2023.^6^ Based on their economic, political and other contextual factors, countries apply different healthcare financing mechanisms including general taxes, social health insurance (SHI), private insurance and out-of-pocket payment (OPP).^7^

SHI got recognition for enabling affordable and equitable healthcare in several high-income countries,^8^ and owing to this success, the WHO commission recommended SHI for risk and resource pooling.^2^ On affirming their commitment to delivering UHC by 2030, many LMICs adopted SHI as their preferred UHC financing strategy. While some evidence^8^ suggests that SHI increases coverage, other evidence indicate a shift from payroll-based to general revenue–based mechanisms for financing health across countries in Europe where the SHI first started.^9^ This reverse direction by the Western world suggests a failure of the SHI to provide sustainable long-term health financing solutions. It therefore begs the question as to whether the SHI rush across LMICs is a misdirection that should be reassessed if LMICs are to make any progress towards universal coverage. This study sought to analyse the potential of the SHI to autonomously finance UHC in LMICs through a policy analysis case study of Kenya. This work is especially important given the ongoing policy reforms of the Kenyan health system and health financing to beat the 2030 timeline for UHC attainment. The first section reviews the literature on SHI in LMICs and thereafter follows a summarised background of Kenya’s health system.

### The state of the art on social health insurance in high- and low-income countries

SHI systems originated in 1883, during the Bismarckian times in Germany, when employers contributed to employee’s sickness funds to avoid workers’ absence due to illness or low productivity due to poor health.^10^ Decades later, these contributions were made compulsory and eventually fully evolved into the SHI and spread to other countries in Western Europe and to the rest of the world as a way to facilitate public funding of healthcare services.^10^

SHI models are a contribution based and ensure a large central pool of funds which provides bargaining power in the purchasing of healthcare services. Unlike general taxes, the resources are earmarked to health thus not distributed across other public sector budget areas. SHI is based on social solidarity and modelled to enable equity in healthcare through subsidisation and cross-subsidisation; contributions are made to the fund regardless of need and used to purchase healthcare regardless of one’s level of contribution or ability to pay.^7 11^ This separates SHI from other private insurance which afford healthcare to only those who pay agreed premiums for a pre-agreed amount of cover. However, SHI systems have some structural limitations. Compulsory payroll-based contributions might seem like indirect tax to the public. Membership and consequently healthcare access are dependent on contribution, therefore leaving a large proportion of the informal and unemployed population exposed to poor health states and financial catastrophe,^10^ unless covered by social welfare policies. Furthermore, payroll-based systems are socially regressive, as the lowest-paid employees contribute a bigger proportion of their employment income, compared with those in higher pay scales. SHI can therefore widen the inequity gap and cause adverse impacts on the economy, especially in LMICs.^12^

Asia, China and Vietnam adopted a compulsory SHI for civil servants and voluntary for the informal sector. They lowered the amount of premium to attract a larger contribution pool, but the unintended consequence was reduced risk protection for the entire population.^8 13^ The social solidarity concept of cross-subsidisation on which SHI is based was not fully accepted in China, especially in areas where incomes were highly unequal. This was majorly due to varying beliefs about poverty, among them being as a result of laziness as opposed to unequal access, among other things. Additionally, there was little to no engagement with the informal and unemployed members of society, which greatly hindered their participation in the coproduction of the SHI policy in China.^5 13^ Additionally, Savedoff^14^ rightly observes that cross-subsidisation, as is intended in SHI, is not often adequately achieved due to the benefits packages attached to SHI. Health services provided by most benefit systems are normally foreseen and commonly demanded by the population and thus are not of insurable risk, leading to high premiums. Moreover, LMICs have fundamental limitations including inefficiencies in public health spending, a lack of political goodwill and poor governance which not only hinder any potential for a successful SHI, but can also increase health inequities and allow wastage of health resources.^14^

The proportion of the informal sector and their inclusion into the SHI model remains to be a glaring hindrance to the success of SHI in LMICs.^15^,^17^ Efforts to have informal sector workers join voluntarily proved difficult, owing to challenges with regulating the informal sector, irregular and unpredictable informal sector incomes which made it difficult to consistently and timely contribute to the SHI (ibid). The informal sector participation in the Philippines was comparatively scaled up by subsidising premiums through informal sector corporate societies and offering flexible repayment terms.^5^ However, other evidence suggest that by excluding populations that are most likely in need of healthcare services, SHI encourages inequity and hinders the UHC progress in LMICs.^12^

In their assessment of labour-based systems of financing healthcare, Yazbeck *et al*^18^ argue that general revenues may be a more sustainable and efficient means of financing healthcare than mechanisms that are based on employment status such as the SHI, and as such, LMICs aiming at achieving UHC should reassess their health financing choices. Additionally, reliance on labour-based systems provides an avenue for governments to defer the liability of paying for healthcare from the government to the few employed members of the population^14^ such as in Thailand and Mexico where the ratio of social security spending per capita was at a point two and five times, respectively, more than the government spending on health.^14^ Moreover, Wagstaff^9^ highlights practices of evasion even within the formal sector using evidence from Vietnam, China and the Philippines where formal sector enrolment was found to be just about 60–75%. This evidence suggests that the capacity to collect revenue through SHI systems is hugely limited, not only in the informal but within the formal sector as well.

#### The relevance and conditionalities of social health insurance (SHI) to achieve universal health coverage (UHC)

UHC may not be attainable through exclusive SHI financing, especially for resource scarce LMICs. Germany, though a high-income nation, took a whole century to attain universal coverage.^10^ South Korea recorded among the shortest timelines (26 years) after introducing mandatory contributions for everyone in the last 11 years before UHC attainment.^19^ Notably, the enforcement of mandatory contributions was proliferated by the set of enabling economic and political conditions that prevailed at the time, a booming economy in the 1980s, which allowed the government fiscal space to enable the subsidisation of premiums and job creation, improving household revenues and, consequently, premium contributions. Furthermore, to increase political support and legitimacy, the government of the day proposed the introduction of UHC for the self-employed ahead of the 1987 elections.^8 19^

In Latin America, Argentina, Brazil, Colombia and Mexico achieved over three quarters essential service coverage (76–77%) and financial protection through complementary tripartite or dual financing systems (public, social security and private insurance).^20^ In addition to contributions by the employed populations, general taxes and other government funds are used to finance healthcare for at-risk and low-income populations, strengthen primary healthcare, subsidise contributions for the unemployed and establish health protection systems to cover the poor and informal sector population.^20^ Similarly in Ghana, a proportion of value added tax was earmarked to co-finance premiums for the poor and vulnerable and the informal population, leading to 75% coverage.^15^

### Kenya’s health system and financing

Kenya is an LMIC democratic republic located in Sub-Saharan Africa, with a population of approximately 55 864 655 million as of 2019, about 30% of whom live below the $1.90 a day poverty line.^21 22^ As of 2020, Kenya’s gross domestic product (GDP) per capita stood at $1838.21, with a health expenditure of 4.59% of the GDP. The health professional density was 0.16 physicians and 1.166 nurses and midwives per 1000 people, and hospital beds were 1.4 units per 1000 population.^23^

Kenya has a devolved governance system consisting of the national government, and 47 counties, to which some public services, including health, are devolved.^24^ The country runs a free healthcare market, comprising both private and public healthcare providers who have equal opportunities to provide healthcare. Public facilities are further classified into six levels: community facilities, dispensaries, health centres, county hospitals, county referral hospitals and national referral hospitals, in order of size and complexity of services offered. The first three provide primary healthcare services, whereas county and national referral hospitals handle curative and other secondary health services.^24^ As of 2021, only about 25% of the country’s population had any form of insurance, implying that about 75% either paid OPP and/or were at risk of health-related financial catastrophes. OPP account for the highest proportion of health expenditure at 26%, followed by the SHI at 17%.^25^

Kenya’s SHI system, the National Health Insurance Fund (NHIF), formerly National *‘*Hospital’ Insurance Fund, was established in 1966 as a state department under the Ministry of Health with a mandate to provide a medical insurance scheme for government employees.^26^ The NHIF has seen several reforms over the years, with the majority happening within the last decade, after the introduction of UHC to the national agenda, and the adoption of NHIF as the main UHC financing mechanism.^2527^,^29^

Kenya’s NHIF, like other SHI models, is employment-based. The employment structure in Kenya consists of about 83% of workers from the informal sector and 17% from the formal sector.^30^ The informal sector is characterised by few to no regulations owing to the irregular and insecure nature of jobs. Most informal income go unaccounted for due to a lack of efficient systems and structures to assess them.^31^ Resultantly, as observed with SHI models in other LMICs, the inclusion of the informal sector into the NHIF has been slow and not devoid of setbacks. In 2021, membership in the NHIF was made mandatory for all residents of the country to compel the inclusion of the informal sector;^32^ however, this reform could not be affected due to the practicalities around it. In 2023, four new complimentary bills were signed to law: the Social Health Insurance Fund (SHIF) Act, Primary Health Care Act, Digital Health Act and Facility Improvement Financing Act.

With the push for UHC attainment, Kenya’s health financing system has been the subject of several rich studies, including analyses of the purchasing reforms, assessment of informal and poor populations participation, reviews of the NHIF reforms and their implication for UHC, among others.^1725 28 33^,^37^ However, a broader comprehensive policy analysis of the NHIF’s content, context, processes and an in-depth analysis of key actors’ interaction with the various policy segments within the policy environment remains undone. Our work aimed to advance this and contribute to the existing body of literature by providing a broad-view policy analysis of the SHI in Kenya and give perspective on the factors within the policy environment that influence the formation, development and implementation of the SHI financing policy in Kenya and other LMICs.

## Methodology

### The research strategy

Through interviews and document analysis, we performed a retrospective analysis of the NHIF to assess why and how the policy was formulated, developed and implemented; what assumptions it was based on; and its consequential impact on the population.^38^ We employed a relativist research approach to analyse how actors’ behaviour and perceptions interplay with the other dimensions of policymaking to influence health policies and outcomes.^39^

We adapted Walt and Gilson’s health policy triangle to analyse Kenya’s SHI.^40^ The policy triangle allows for a comprehensive analysis by enabling the inclusion of actors and their interplay with the policy contents and processes within a given context (ibid). This framework was, therefore, appropriate for our analysis of the content and process of designing, developing and implementing Kenya’s SHI and rationalising the involvement of key stakeholders within the health system. The concept of the policy triangle has great relevance for Kenya, having been originally designed to facilitate policy analysis and provide guidance on the implementation of health reforms in the rise of health systems crises in LMICs.^40^ To effectively transfer the concept, we made various adaptations to contextualise the original policy triangle to Kenya’s policy environment.^41^

### Adaptation of the policy triangle

A policy context comprises a set of situational, structural or cultural factors, otherwise categorised as social, political or economic factors that influence health policy.^42^ We contextualised the policy environment by considering the structural and situational factors that influence policymaking in Kenya.^41 42^ We investigated context-specific social, political and economic factors that influenced the formulation, development and implementation of the NHIF.

The analysis of the policy process involved looking into the formulation, development and implementation of the SHI policy.^42^ Given the non-linear development of the SHI, the process analysis slightly deviated from the usual heuristic model which begins from problem identification, through to implementation.^43^ Rather, we performed a chronological review of the historical account of the NHIF, highlighting significant developments and reforms that the fund had undergone since its inception as a ministry department, through to the SHI agenda setting and ultimately its current role as the main financing mechanism for UHC in Kenya.

The analysis of the policy content was done systematically. We compartmentalised the content into three broad sections namely premiums, benefits packages, and claims and reimbursements. We then analysed the functionality of the content of each compartment independently.

**Figure 1:**
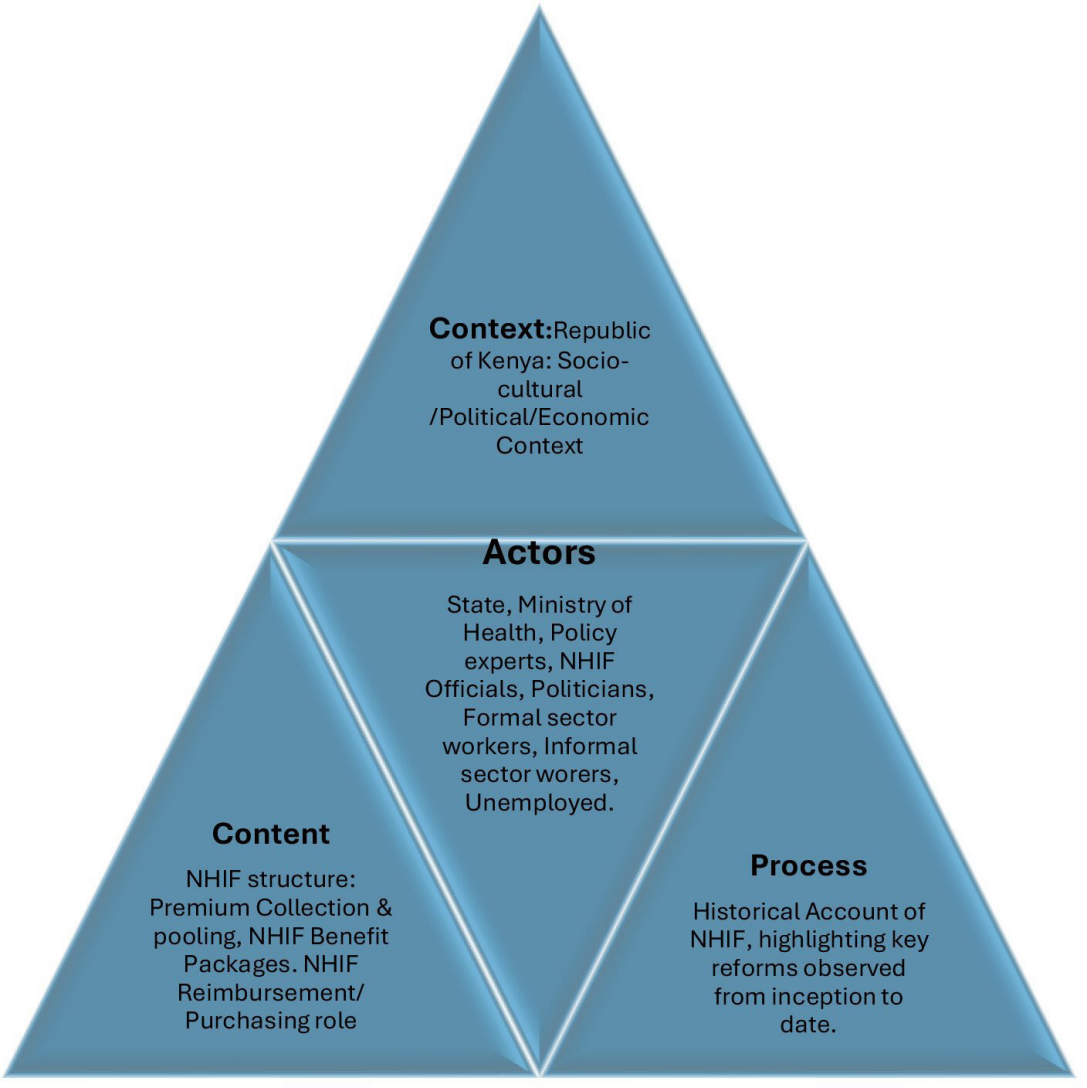
Adaptation of the Gilson and Walt (1996) policy triangle to study the social health insurance development in Kenya. Each triangle represents a dimension for policy analysis: context, content, processes and actors. It includes the physical, political, socioeconomic and cultural contexts; the policy content including the structure, revenue collection and pooling, the benefit packages, and payment and purchasing. It also includes the policy reform process and the array of actors involved in the different dimensions of the policy. NHIF, National Health Insurance Fund.

Finally, the actor analysis, which was the most vital part of the study, sought to establish the engagement and perceptions of key actors. Proposals for the inclusion of diverse actors in policymaking and analysis have increased over the recent past, with some authors challenging the top-down bureaucratic prioritisation of actors.^41 42^ In our adaptation, we included an array of actor representatives to reflect an actor-network beyond just the state and top political decision-makers. Our actor analysis included the state, Ministry of Health officials, national and private sector health policy experts, NHIF officials, formal and informal sector workers, and the unemployed (figure 1).

### Data collection

We used three distinct methods to collect data for our analysis: document analysis, financial database analysis and interviews. Our choice of methods was guided by the sections of the policy triangle and the availability of data for each part. We conducted a review of documents, literature reviews and review of financial reports and audited NHIF accounts to obtain data on the policy content, process, context as well as the actors’ engagement. Thereafter, we conducted in-depth interviews to clarify and verify our observations from the document reviews regarding the four components of the policy triangle and to obtain the key actors’ perceptions on the predictors of the SHI.

#### Documental analysis

We applied a search strategy to identify documents to be included in the review. A general search was done on Google, with the keywords being ‘NHIF Kenya Policy and Procedure’. Different other keywords were applied to the search engine, including ‘NHIF Kenya reforms’ and ‘social health insurance Kenya’. The initial search yielded 63 600 results, including policy briefs, publications, Ministry of Health guidelines, NHIF publications, scholarly articles, bills and acts of parliament, financial reports and audited NHIF accounts, reports from non-governmental organisations, blog posts and other grey literature. The titles of the articles yielded by the searches were screened for relevance and duplicates, leaving 46 that met the inclusion criteria. These documents were reviewed, and their reference lists were also checked to obtain additional relevant documents. Grey literature such as mainstream newspapers, ‘The Standard’ and ‘Daily Newspaper’, were also reviewed.

#### Inclusion and exclusion criteria

The inclusion criteria for the document selection was that the articles had to be officially published reports from Kenya, official ministry guidelines, acts of parliament or NHIF official publications. Unofficial reports, policy briefs and commentaries on Kenya’s NHIF, blog posts and social media posts were excluded from the final document analysis.

#### Interviews

To establish the engagement and perceptions of actors in the NHIF policy, interviews were conducted with key stakeholders including health systems policy experts, private and public healthcare providers, NHIF officials, ministry officials and members of the public drawn from the formal and informal sector. Through purposive sampling and snowballing, a total of 25 participants were recruited for the study, and 21 in-depth interviews were conducted successfully. Our interviews covered a broad range of stakeholders including five policymakers, nine key informants (Ministry of Health officials, NHIF officials, public and private healthcare workers and hospital clerks) and seven members from the formal and informal sector (online supplemental file 1). Interviews were conducted online via Zoom and were recorded using an end-to-end encrypted audio recorder. When there seemed to be no more new information, the primary researcher assumed the saturation point, and data collection was stopped.^44^ Data were stored in password-secured files for confidentiality.

### Data analysis

All the interviews were recorded and transcribed in English. The audio recordings from the interviews were then transcribed using an MS Word voice typing app. All interview participants were assigned name codes to ensure anonymity. The name-coding was based on their engagement with the NHIF and included codes such as ‘private healthcare provider-001’, ‘public healthcare provider-002’ and ‘health systems expert-003’. Data from the document analysis and the interview transcription were fed into an Excel sheet and color-coded to represent either interview data or document review data. We employed the thematic approach to our data analysis.^45^ Themes and subthemes were identified across both data sets. All data were analysed fairly, and all common themes emerging from interviews with the different stakeholders were triangulated alongside those from document reviews. Common broad themes identified included ‘informal income’, ‘inequality in healthcare access’, ‘fraud and corruption’, ‘political goodwill/lack of’, ‘poverty’ and ‘communication’. The interview data were particularly important for strengthening the credibility and quality of the evidence (see box 1 below).

Box 1 **List of themes identified in the policy analysis**

**From document reviews:**

- Improved coverage and access to healthcare.
- Informal sector enrolment.
- Regressive system.
- Financial consequences; fines.
- Political goodwill and/or lack of.
- Corruption and embezzlement of funds.
- Inequality in access to healthcare.
- Subsidies for the poor and vulnerable.
- Enhanced schemes for some occupation groups.

**From interviews:**

- Poverty, unemployment and irregular informal incomes.
- Misunderstanding about the social health insurance concept.
- Lack of communication; little engagement with stakeholders.
- Fraud; kickbacks.
- Fines for defaulting payments.
- Inequality in access to healthcare.
- Improved coverage and access to healthcare.
- Subsidy for the poor and vulnerable.
- Enhanced schemes for some occupation groups.

### Ethics approval

In compliance with the ethical requirements for conducting primary research, we applied for ethics approval to the Queen Mary University of London Ethics Committee, complete with the research information sheet and the consent form that we would later share with the interviewees. The study was low risk and did not require the ethics committee to convene. After application and following established policy and procedures, approval was obtained (reference number QMERC22.151). Simultaneously, we also obtained ethics approval from the Kenya National Commission for Science, Technology, and Innovation (clearance number: NACOSTI/P/22/16580).

### Patient and public involvement

Patients and the public were not involved in the design, conduct, reporting or dissemination plans of our research.

## Findings

This section presents the findings from interview data and document analyses generated by our policy analysis methodology. We used our adapted framework to extract and organise the findings below. It begins by providing evidence of the informal nature of the labour market in Kenya and its role in the success of SHI. It then proceeds to summarise the NHIF reform process and the concerns of our interviewees on the efficiency of the system’s purchasing and payment mechanisms and an overview of the pros and cons of the available parallel schemes run by the NHIF.

### Informality of the labour market

The evidence collected and the relative analysis found an imbalance of SHI contributions owing to the high proportion of the informal population in the country. Until recently, contributions to the NHIF were compulsory for the formally employed and voluntary for members of the informal sector.^26^

As of 2019, the proportion of informal workers in Kenya stood at 83% of the entire country’s 18.1 million working population. The informal sector NHIF membership thus represented a bare 27.7% uptake within the informal sector.^30 46^ Furthermore, a 2020 national survey further found that only 1% of the country’s informal employers remitted NHIF for their informal workers,^30^ revealing a significant gap in the inclusion of the informal sector workers to the NHIF (table 1).

**Table 1:** NHIF membership by sector (2019)

Category	2019/2020 totals	How premiums are paid
Formal sector	4 439 682	Premiums paid through a salary graduated scale
Informal sector	4 262 354	Base premium Kes 500 ($5) paid monthly by each member
Sponsored programmes	799 049	Premiums paid on behalf of members by the government/non-government organisations/community-based organisations
Total NHIF membership (2019)	9 501 085	Mix of personal and employment-related contributions

Source: Kenya National Bureau of Statistics (2021). National Economic Survey, 2021.

NHIFNational Health Insurance Fund

The graduated scale premium contribution system had the least premium contribution at Kes. 500 ($5) and the highest at Kes. 1700 ($17) for the formal sector, whereas the base premium rate was set at Kes. 500 ($5) for informal members.^26^ The NHIF Amendment Bill, 2021, was required of formal employers to top-up the difference of the minimum premium contributions for their employees whose low salary scales meant lower than the base NHIF premium contribution. No such co-contribution mechanism was put in place for the informal sector members, who regardless of their income levels had to pay the entire base premium amount. Furthermore, the graduated salary scale contribution system was found to be regressive as the least-earning members paid significantly higher proportions of their income to premium contributions, compared with higher-income earners (online supplemental file 2).

Moreover, an analysis of willingness and ability to pay for NHIF premiums among the informal sector revealed that the base monthly premium, Kes 500 ($5), was unaffordable for about 75% of the informal population.^47^ Data obtained from interviews with a section of informal sector workers indicated that health insurance was not considered a primary need given the existence of more pressing basic needs.

*I have so many more immediate needs that I cannot afford to save that KES 500 every month to pay NHIF*. -Informal sector worker-001

The review of the NHIF guidelines further observed that the NHIF had a set date by which premiums were expected to be paid every month, failure to which, members incurred fines.^26^

The fines imposed for missed premium payments were noted to be 50% of the member’s applied premium rate and a suspension from accessing healthcare. Reinstatement of the member and their dependents’ access to healthcare services was noted to be dependent on them paying the entire amount of missed premiums plus the fines accrued, which increased with every missed or late monthly payment.^26^ Through data collected during interviews, the informal sector members reported dissatisfaction with the model by which the penalties were applied. Members argued that often, the reasons for missing payments are usually valid, unintended and not warranting additional financial liability, leading to membership drop-out.

…*Sometimes when you can’t afford it and you miss some months, and you go to the hospital critically ill, you cannot be seen until you pay all the months that you missed plus all the fines…it becomes too expensive, people quit*. -Informal sector worker-002

### The reform process

Our document review observed that the NHIF has undergone several reforms since its inception in 1966. The first and most notable reform was the invitation of the public to the NHIF in 1972.^48^ Membership was acquired by paying a standard monthly contribution to the fund. The contribution was mandatory for formal sector workers and voluntary for everyone else. Nearly two decades later, in 1992, user fees were introduced due to a highly constrained national budget that was inadequate in covering the population’s healthcare expenditure.^33^ This was shortly followed by a graduated scale contribution system, whereby premiums were deducted according to earnings as shown in table 1 below. The next major reform was the upgrading of the NHIF from a ministry department to a state corporation under the NHIF Act No.9 of 1998.^48^

The millennium birthed the SHIF agenda into the country’s political space. Taskforces were formed to develop SHI strategies, and international support was sought for the same. In 2004, the president declined to assent to the SHI recommendations of the WHO/Deutsche Gesellschaft für Internationale Zusammenarbeit (GIZ) expert missions, alluding to sustainability, affordability and technical concerns.^33^ This was followed by nearly a decade-long hiatus in active NHIF reforms. In 2012, the Civil Servants Scheme and other Enhanced schemes were introduced.^48^ Shortly afterwards, in 2015, there was an upward revision of up to 413% of the NHIF premium contributions (see online supplemental file 2) and the NHIF benefit packages.^49^

In 2015, the government of Kenya, like many other states, affirmed its commitment to the WHO to deliver UHC by 2030. This re-ignited the SHI agenda in the country. In 2018, the government declared UHC among its top 4 priority agendas. The NHIF was adopted as the main mechanism for financing UHC. In 2021, membership in the NHIF was made mandatory for all citizens,^32^ and shortly afterwards, in 2023, a new SHIF Act was signed to law to repeal the existing NHIF Act. The SHIF Act introduces a flat rate contribution system (2.75%) of all formal income and is to be augmented by two new government-funded funds: the Primary Healthcare (PHC) Fund and the Emergency, Chronic and Critical Illness (ECCI) Fund. The SHIF Act repeals the NHIF Act and all its mandates and introduces a new corporate body, the Health Insurance Authority, to oversee all operations of the new SHIF.^50^ Table 2 below summarises the major NHIF reforms.

**Table 2:** Major NHIF reforms since inception to date

Year	Significant event
1966	The NHIF was established as a department under the Ministry of Health with the mandate to provide a contributory hospital cover for formally employed citizens earning more than Kes 1000=$10.
1972	Voluntary contribution to the fund was open to members of the informal sector and those earning below Kes 1000=$10.
1989	A healthcare cost-sharing system was introduced in the form of user fees and was abolished in 1990 due to concerns and lobbies on social justice. The national budget was critically constrained and could not sufficiently finance health, leading to reintroduction of user fees in 1992.
1990	A graduated scale contribution system was introduced, and minimum salary subject to contribution was capped at Kes 15 000=$150. This increased the premiums, the lowest being Kes 30=$0.3 and the highest being Kes 320=$3.20
1998	The 1966 NHIF Act which made the fund a department under the Ministry of Health was repealed, and the fund was reinstated to an autonomous state corporation under the NHIF Act of 1998. The aim of this change was to improve the efficiency and relevance of the fund.
2001	The idea of a SHIF was hatched with a strategy of precollecting the out-of-pocket user fees before the need for use arises. The president gave a directive to cabinet ministers to find measures of introducing a mandatory Social Health Insurance System for all Kenyans.
2003	The Economic Recovery Strategy for Wealth and Employment Creation was adopted with the agenda to transform the existing NHIF to a NSHIF. Having received the report from the assigned taskforce, the Ministry of Health approached the WHO/Deutsche Gesellschaft für Technische Zusammenarbeit (GTZ) for technical support to establish a SHIF.
2003–2004	6 expert missions reviewed, suggested amendments and trained on the proposed mandatory SHIF. The bill was discussed and passed by parliament at the end of 2004. However, the presidential assent was declined citing affordability, sustainability and technical concerns.
2015	The NHIF revised the premiums and expanded the benefit package. The revision increased the premiums by up to 431%.
2018	Kenya embarked on a journey to deliver UHC by 2022, championed by the sitting president under his Big Four National Agenda. NHIF was chosen as the financing mechanism to deliver UHC.
2021	Parliament passes a bill to make NHIF mandatory for all citizens.
2023	A new SHIF is established to annul the NHIF Act, and a new corporate body, the Social Health Authority, is established to oversee all operations of the SHIF, effective March 2024.The SHIF introduces flat rate contributions of 2.75% of all formal income and is to be supplemented by two new government funded funds: the Primary Healthcare Fund and the Emergency, Chronic and Critical Illness Fund.

Source: NHIF Profile, 2023; NHIF Act, 1998; GTZ/WHO, 2006; NHIF Amendment Act, 2022; Social Health Insurance Act, 2023.

NHIFNational Health Insurance FundNSHIFNational Social Health Insurance FundSHIFSocial Health Insurance Fund

Stakeholders shared divergent views on the lates reform that made NHIF mandatory for all. Some agreed that the regulation is only theoretical and more practical measures would need to be employed.

*The policy is now making it mandatory for those in the informal employment; however, there is still laxity among this group. The fund needs to make more efforts to actually reach them*. - National policy expert-001

Others expressed optimism that the mandatory contributions would be beneficial in ensuring consistency of contributions, thus providing a stable financial pool.

*NHIF Act having been amended to require a waiting period for claims is now more aligned with the fact that majority of the targeted pool is from the informal sector. Before, informal sector subscribers would only pay NHIF when they know they’ll need healthcare and stop paying after they received care, which would make the fund unsustainable*. - MoH Official-001

### Inefficiency in purchasing and payment

Data collected from interviews with policy experts and healthcare providers reported inefficiency in the purchasing and payment functions of the NHIF. The purchasing rates for public facilities were reported to be insufficient and incapable of delivering the promised health benefits for patients.

*The reimbursement rules are not aligned with sustainable UHC as there has to be changes in other factors to make healthcare providers be able to provide quality healthcare at the reimbursement rates provided for in the Act*. - MoH Official-003

Political interference and conflict of interests was reported to play a major role in the misalignment of healthcare purchasing.

*Politicians push for lower and lower premiums that make the fund put out impracticable reimbursement rates for healthcare providers*. -National policy expert-003

In addition to bureaucracy and fraud in the claims and reimbursement processes of the NHIF, corruption was reported to occur across the arms of the health system and was perpetrated by key actors within the system, including government and NHIF officials, healthcare providers and the healthcare users.

*Some providers claim that they have done surgeries that they haven’t and the documents are doctored in a way to show that they provided a service that indeed they did not provide*. MoH Official-001

The interviews also revealed collusion between some NHIF staff and healthcare providers for kickbacks in exchange for faster processing of reimbursements, especially with private facilities, which ultimately had their claims timely and sufficiently reimbursed.

*NHIF colluding with the providers to give kickbacks when they are paying for claims. People create bureaucracies within the system, or they make it difficult to investigate that quality in a way that makes it easy to collude with those providers*. - MoH Official-002

Healthcare providers in public facilities, on the other hand, reported frustration during reimbursement. Facilities in rural areas particularly reported deplorable working conditions including lack of basic medical and administrative supplies, staff demoralisation and consequently compromised quality healthcare given to patients.

*Reimbursement for services is very frustrating for public hospitals especially… the attitude is like why should the government give to the government?* Private/Public Healthcare Provider-001

*I work in a very resource poor setting, thepatientsI see are registered with NHIF, yet I do not have even the most basic of drugs likeIVor paracetamol*. - Public Healthcare Provider-001

### Parallel schemes

The review found several occupation-based enhanced schemes under the NHIF, where employers paid extra premium beyond their employees’ individual NHIF contributions in exchange for lucrative medical benefit packages (table 3).^26 51^

**Table 3:** Kenya’s NHIF schemes

NHIF schemes	Membership	Premium contribution
National Scheme(Supa Cover): Standard	Open to every resident of the country and their dependants.	Premiums worked on a graduated scale for formal sector; $5 for informal sector.
Civil Servants’ Scheme(Enhanced)	Civil servants and their dependants (one declared spouse and up to five children of ages 0–21 years and up to 25 years old if in fulltime formal education).	Employee premium+additional paid by employer=enhanced benefit packages.
County Government Scheme(Enhanced)	Employees of county governments which have contracted NHIF+their dependants (one declared spouse and up to five children of ages 0–21 years and up to 25 years old if in fulltime formal education).	Employee premium+additional paid by employer=enhanced benefit packages.
Government Parastatals(Enhanced)	Employees of government parastatals which have contracted NHIF+their dependants (one declared spouse and up to five children of ages 0–21 years and up to 25 years old if in fulltime formal education).	Employee premium+additional paid by employer=enhanced benefit packages.
National Police and Prisons Service(Enhanced)	Members of the national police and prisons service, and their dependants (one declared spouse and up to five children of ages 0–21 years and up to 25 years old if in fulltime formal education).	Employee premium+additional paid by employer=enhanced benefit packages.
EduAfya Scheme(Standard)	All students in public secondary schools registered under the National Education Management Information System.	NHIF funded by the ministry of education for all children enrolled in public high schools. The students do not contribute.
Health Insurance Subsidy for the Poor (HISP) Scheme(Subsidy)	Households of the indigents, older persons and the disabled in Kenya.Beneficiaries get the standard national benefit package.	Premiums are fully paid by the government. Beneficiaries do not contribute.
Linda Mama Free Maternity Scheme(Subsidy)	All pregnant women who are residents of the country, expires 6 months after delivery and covers the babies until age 5.	Premiums are fully paid by the government. Beneficiaries do not contribute.

Source: NHIF Handbook for Comprehensive Medical Cover for Civil Servants, National Police, County Governments, Public and Private Institutions, 2015. NHIF Subsidy Programs for UHC, 2021.

NHIFNational Health Insurance Fund

Review of the enhanced covers handbook found that the NHIF provided comprehensive benefit packages for various enhanced schemes. The enhanced schemes promoted enrolment among the biggest public sector employers and expanded the resource pool. However, a comparative review of the benefit packages found significant differences between the enhanced covers and the national cover, including dental and optical care, air ambulance, overseas treatment and last expense cover, among several others. Members of the enhanced schemes were also noted to have more options of healthcare facilities to choose from, as opposed to members of the national cover who were capitated to obtain healthcare only at their specified facilities.^26 51^

The review of NHIF premium collection and pay-out rates between the national scheme and the enhanced covers observed no cross-subsidisation between the schemes. The pay-out on the national scheme was 95.89% of the scheme’s total contributions, indicating no cross-subsidisation from the enhanced to the national scheme (online supplemental file 3).

Data obtained from interviews indicated that healthcare providers preferred cash paying or privately insured patients because the NHIF reimbursement process was feared to be complicated.

*Some facilities do not even claim for that money…sometimes it is lack of knowhow by the hospital clerks because NHIF has a lot of paperwork and if you don’t do it properly you don’t get the funds*. - Private/Public Healthcare Provider-002

Other than the enhanced schemes, our document reviews found subsidy schemes paid by the government to cover various vulnerable population groups.^52^ The first cover was the maternity cover dubbed ‘Linda Mama’, under which all pregnant women in the country are eligible for accessing quality prenatal, delivery and postdelivery healthcare. The older persons and persons with disability subsidy programme was reported to cover about 42 000 households, whereas the Health Insurance Subsidy Program (HISP) that was meant to increase social protection covered 1 million poor families in the first year of its official rollout.^52^

Our review of the HISP handbook found that households included in the subsidy programmes were reported to be obtained from the Ministry of Labour and Social Protection databases and were beneficiaries of the government cash transfer social protection programmes.^52 53^ The programme aimed to cover the populations least 10%. As at the time of its roll-out, about 30% of the population lived below the poverty line, while an estimated 9% lived in extreme poverty.^21 52^ Furthermore, despite having started programme piloting in 2016, and having its official roll-out in 2021, the review found no clear scalability and sustainability plan for the subsidy scheme.

*The government through the exchequer paid NHIF for about1 millionpoor and vulnerable families during the last financial year…the programme has just been rolled out and it is up to the next government to decide how they will go about it*. - MoH official-002

### Political influence, stakeholders’ engagement and communication with other relevant constituencies

Key stakeholder interviews revealed a lack of sufficient engagement with relevant stakeholders in the policymaking and implementation processes. We observed that in some instances where stakeholders were engaged, suggestions were either shelved or not passed in parliament.

*There was a health benefits advisory panel that was constituted by the minister for health in2018,but the findings of this panel were never made public and not adopted by NHIF*. -National policy expert-001

Even though stakeholders are often involved in drafting and reviewing the policies, there is always limited time for participation…in some instances where this is done, the policies are not passed in parliament. - National policy expert-002

Key stakeholders pointed to the Constitution of Kenya 2010, the President’s Big4Agenda 2018, Vision 2030 and the Health Act 2017 as the major enablers of NHIF. Political goodwill was thus observed to be particularly essential for the formulation, development and successful implementation of the NHIF policy.

…*Accelerating the attainment of UHC is one of the best gifts the legislators could have given their electorate*… - Cabinet Secretary for Health (People’s daily newspaper)

## Discussion

This study provides a detailed policy analysis of the implementation process of the SHI system in Kenya, using documental analysis evidence and perceptions from stakeholder interviews. Our findings indicate that although the SHI is perceived as having the capacity to increase healthcare coverage, various challenges continue to impede its potential to finance UHC in Kenya. We gathered evidence that the structure of the country’s predominantly informal job market may represent the main challenge for the implementation of the SHI. We also identified significant inefficiencies in the purchasing and payment of healthcare services. We found evidence that the policy reform process in Kenya remains highly politicised, with opportunities for reforms and the uptake of experts’ advice heavily dependent on political goodwill. Lastly, our analysis uncovered equity gaps in the content and implementation of the NHIF reform, which should be tackled for the success of the new SHIF.

This study offers an original empirical angle to explore the suitability of social insurance financing mechanisms for UHC advancement. However, our findings are subject to a few limitations that qualify their interpretation and validity for other contexts. First, the number of participants interviewed was limited, and a larger sample might provide a more complete representation of Kenya’s health system stakeholders. Second, due to the narrow political window within which the study was carried out, we were unable to include some historical political actors who back in the day played a role in the SHI policy process. Future research should expand the scope of stakeholders to obtain a more global perception of the SHI in Kenya and similar contexts.

Our major finding was that the predominantly informal sector (83%) hindered the expansion of the SHI pool due to its low uptake and inconsistent contributions to the NHIF.^30^ This finding was consistent with previous observations from Kenya, China, Ghana, Colombia, Thailand and other developing countries,^515^,^17 25 33 37 54 55^ which labelled informal sector participation as the biggest challenge of the SHI. Like many other SHIs, contributions to the NHIF have been mandatory for the formal workers and voluntary for the informal sector and the rest of the population, implying a small contributory base that is unlikely to sufficiently finance UHC. Based on the evidence from other countries that have made significant progress to UHC through co-financing their SHI with other government-funded options,^15 20^ co-financing mechanisms that cover the informal population, the poor and vulnerable residents should be improved to supplement the SHI. The new SHIF which proposes registration for all residents of the country, and a flat rate contribution of 2.75% of all formal income, supplemented by additional earmarked government funds promises a broader pool for UHC financing in Kenya.

Our findings pointed to inefficiencies due to wastage, misappropriation, bureaucracy and fraud within the health system causing major hurdles for UHC financing. Inefficiency claims were mainly observed in the purchasing and payment of healthcare. The NHIF was noted to have more pay-outs to private hospitals, mostly facilitated with collusion between staff for kickbacks in exchange for swift processing of claims. On the other hand, some public facilities lacked basic supplies due to slow bureaucratic claims and reimbursement processes. Our findings confirmed previous analysis of the purchasing reforms in Kenya,^35 36^ which highlighted weak strategic purchasing framework and accountability concerns with the implementation of the reform, impacting negatively on the efficiency of the fund. The malpractices in the management of the fund presented a cause for worry causing mistrust among members of the fund and consequently demotivating participation. SHI is anchored on socialism, trust and solidarity, all of which lead up to the success of cross-subsidisation that SHI heavily relies on.^10^ The fund therefore needs to remain credible and accountable to retain social support which is paramount for its success. In addition to implementing SHI, health systems should implement strategic purchasing mechanisms and reinforce measures to ensure accountability and transparency to curb the inefficiencies, bureaucracies and misappropriation observed in the purchasing and payment system.

Policy formulation and implementation in Kenya are highly influenced by political actors and the schedule of political events; although this is far from uncommon in the Sub-Saharan context,^56^ we believe this poses a particularly heavy burden for the achievement of UHC in Kenya. The initial SHI agenda setting was instigated in 2004, and two decades later, characterised by government transitions, a long impasse followed by a myriad of reforms in the last decade,^25 28 33 35 36 55^ Kenya is yet to establish a fully functional SHI. The recent reform to impound the NHIF and establish a fresh SHIF and supplementary PHC and ECCI funds are positive indications of the country’s commitment to UHC; however, their success remains subject to continued political goodwill and efficient implementation.

Finally, eminent inequity was observed in the imposition of fines of up to 50% of the premium, on members who default on their premium contributions. Such members were in addition, barred from accessing healthcare unless they paid OOP.^57^ We argue that this strategy is impractical for low-income and informal populations whose income is known to be irregular and unpredictable. Such administration of penalties and hard lines increases the equity gap and contradicts the intention of the SHI and the goal of UHC, which is to provide health gains to all people, regardless of their ability to pay.^1 3^ Additionally, the several schemes run by the NHIF propagated inequity through the provision of different cover/benefit packages determined by the premium amount paid. This worked contrary to the UHC goal to ‘provide all people with access to needed health services’ implying universality and equity.^3^

### Recommendations for policy

If confirmed, our findings indicate ways in which the current health financing reform process may be made more equitable. First, the fund should devise ways of including informal occupation groups, such as incentivising premiums to cooperative societies affiliated with informal occupation groups to enable bulk payment of members’ premiums on flexible repayment terms. This system was successful in expanding coverage to the informal sector in the Philippines.^5^ Second, the introduction of the flat rate premiums and earmarked supplementary funds: the PHC fund and the ECCI fund are a noteworthy move whose efficiency and success will be dependent on the transparency and accountability of the new Social Health Authority. There is need for use of complex information technology systems which facilitate efficiency by highlighting gaps and bottlenecks that may exist across different operational activities. Regular monitoring and evaluation of the fund are also needful to keep all actors accountable and efficient.

Thirdly, political goodwill and public support of the SHIF should be encouraged. The fund should promote better engagement with the public and key stakeholders and improve the uptake of expert advice. Political goodwill has been observed to be a cornerstone for the establishment of a successful SHI in both high-income and low-income countries.^5 13 19^ Finally, all stakeholders are to recentre their focus on health equity as a component of UHC and the SHI financing mechanism. As much as possible, all decisions need to be weighed against their equity considerations, to lessen equity gaps in healthcare, and ensure that healthcare access is indeed universal affordable and equitable.^58^,^60^

## Conclusions

SHI can provide a path for financing healthcare and improving coverage in LMICs. Although significantly limited by its labour-based nature and other structural and contextual challenges, the risk minimisation and resource pooling properties of the SHI allow for cross-subsidisation of services, which, if well executed, can improve healthcare access. This study conducted a retrospective policy analysis of Kenya’s SHI model, through interviews with key stakeholders and document analysis.

Although the SHI system is perceived to increase coverage and the quality of Kenya’s health services, substantial structural and contextual challenges appear to hinder its potential to finance the attainment of UHC, which is just a stepping stone towards comprehensive healthcare attainment. From Kenya’s experience, we identify informal sector participation, inefficiency of purchasing and payment of healthcare, and political engagement, as key bottlenecks which if tackled correctly, the SHI could benefit LMICs. LMICs adopting SHI need to also implement co-financing arrangements that do not impose on the population to co-finance, strategic purchasing systems, political goodwill, and good governance for the SHI systems to be beneficial. The newly accented reform introducing the SHIF, and the additional supplementary funds remains a subject for future analysis.

## supplementary material

10.1136/bmjopen-2024-085903 online supplemental file 1

10.1136/bmjopen-2024-085903 online supplemental file 2

10.1136/bmjopen-2024-085903 online supplemental file 3

## Data Availability

All data relevant to the study are included in the article or uploaded as supplementary information.
