# Supplementary material for: Social Health Insurance for Universal Health Coverage in Low and Middle-Income Countries (LMICs): a retrospective policy analysis of attainments, setbacks and equity implications of Kenya’s social health insurance model
Source: BMJ Open. 2024 Dec 11;14(12):e085903. doi: 10.1136/bmjopen-2024-085903 (PMC11647346; doi:10.1136/bmjopen-2024-085903)
Supplement: online supplemental file 1 [file bmjopen-14-12-s001.docx]

Annex 1: List of anonymised interviewees, by function.

| No. | Interview Group | Interviewee (name coded by professional category) | Policy Component Covered During the Interview |
| --- | --- | --- | --- |
| 1 | Key informant | MOH official-001 | Content, processes, context, actors |
| 2 | Key informant/Policy maker | MOH official-002 | Content, processes, context, actors |
| 3 | Key informant/Policy maker | National policy expert-001 | Content, processes, context, actors |
| 4 | Policy maker | National policy expert-002 | Content, processes, context, actors |
| 5 | Policy maker | National policy expert-003 | Content, processes, context, actors |
| 6 | Key informant | Private sector health policy expert-001 | Content, processes, context, actors |
| 7 | Key informant | Private sector health policy expert-002 | Content, processes, context, actors |
| 8 | Key informant | NHIF official-001 | Content, processes |
| 9 | Key informant | County hospital clerk-001 | Content, processes |
| 10 | Key informant | Public healthcare provider-001 | Content, processes |
| 11 | Key informant | Public healthcare provider-002 | Content, processes |
| 12 | Key informant | Private healthcare provider-001 | Content, processes |
| 13 | Key informant | Private healthcare provider-002 | Content, processes |
| 14 | Key informant | Public/Private healthcare provider-001 | Content, processes |
| 15 | NHIF Member (Civil Servants Scheme) | Civil Servant (County government staff)-001 | Content |
| 16 | NHIF Member (Civil Servants Scheme) | Civil Servant (National police service)-001 | Content |
| 17 | NHIF Member (Formal sector, National Scheme) | Private sector (formal) employer-001 | Content |
| 18 | NHIF Member (Formal sector, National Scheme) | Private sector (formal)worker-001 | Content |
| 19 | Non-NHIF Member (Informal sector) | Informal sector worker-001 | Content |
| 20 | NHIF Member (Informal sector, National Scheme) | Informal sector worker-002 | Content |
| 21 | Non NHIF member (Informal sector) | Unemployed youth-001 | Content |
