# Supplementary material for: Social Health Insurance for Universal Health Coverage in Low and Middle-Income Countries (LMICs): a retrospective policy analysis of attainments, setbacks and equity implications of Kenya’s social health insurance model
Source: BMJ Open. 2024 Dec 11;14(12):e085903. doi: 10.1136/bmjopen-2024-085903 (PMC11647346; doi:10.1136/bmjopen-2024-085903)
Supplement: online supplemental file 2 [file bmjopen-14-12-s002.docx]

Annex 2: NHIF premiums salary graduated scale (before and after reforms)

| Sector | Salary Scale | Monthly Contribution 1990-2015 | Monthly Contribution 2015-Date / and proportion of income | |
| --- | --- | --- | --- | --- |
| Formal Sector Employees | KES 1,000-5999 ($10-59) | KES 30-120 ($0.30-1.20) | KES 150 ($1.50) | |
|  | KES 6,000-7999 ($60-79) | KES 140-160 ($1.40-1.60) | KES 300 ($3) | |
|  | KES 8,000-11,999 ($80-119) | KES 180-240 ($1.80-2.40) | KES 400 ($4) | |
|  | KES 12,000-14,999 ($120-149) | KES 260-300 ($2.60-30) | KES 500 ($5) | 4.16% |
|  | KES 15,000-19,000 ($150-190) | KES 320 ($3.20) | KES 600 ($6) | 4.00% |
|  | KES 20,000-24,999 ($200-249.99) |  | KES 750 ($7.50) | 3.75% |
|  | KES 25,000-29,999 ($250-299.99) |  | KES 850 ($8.50) | 3.40% |
|  | KES 30,000-34,999 ($300-349.99) |  | KES 900 ($9) | 3.00% |
|  | KES 35,000-39,999 ($350-399.99) |  | KES 950 ($95) | 2.71% |
|  | KES 40,000-44,999 ($400-449.99) |  | KES 1,000 ($100) | 2.50% |
|  | KES 45,000-49,999 ($450-499.99) |  | KES 1,100 ($110) | 2.44% |
|  | KES 50,000-59,999 ($500-599.99) |  | KES 1,200 ($120) | 2.40% |
|  | KES 60,000-69,999 ($600-699.99) |  | KES 1,300 ($130) | 2.17% |
|  | KES 70,000-79,999 ($700-799.99) |  | KES 1,400 ($140) | 2.00% |
|  | KES 80,000-84,999 ($800-849.99) |  | KES 1,500 ($150) | 1.88% |
|  | KES 90,000-99,999 ($900-999.99) |  | KES 1,600 ($160) | 1.78% |
|  | KES 100,000 ($1,000) and above |  | KES 1,700 ($170) | 1.70% |
| Informal Sector | Flat rate for all informal sector wages and unemployed members | KES 160 ($1.60) | KES 500 ($5) | |

*Source: National Health Insurance Fund, 2023*
