# Supplementary material for: Social Health Insurance for Universal Health Coverage in Low and Middle-Income Countries (LMICs): a retrospective policy analysis of attainments, setbacks and equity implications of Kenya’s social health insurance model
Source: BMJ Open. 2024 Dec 11;14(12):e085903. doi: 10.1136/bmjopen-2024-085903 (PMC11647346; doi:10.1136/bmjopen-2024-085903)
Supplement: online supplemental file 3 [file bmjopen-14-12-s003.docx]

Annex 3. NHIF Annual Premium Contributions & Benefit Pay-out for Year 2019/20

| **Key payments** | **National Scheme** | **Enhanced Schemes** |
| --- | --- | --- |
| Premium Contribution | 31,254.6 | 28,245.5 |
| Benefit Pay-out | 29,971.1 | 24,391.5 |
| Pay-out Ratio | 95.89% | 86.36% |

*Source: Kenya National Bureau of Statistics. Economic Survey, 2021*
