# Supplementary material for: Social Health Insurance for Universal Health Coverage in Low and Middle-Income Countries (LMICs): a retrospective policy analysis of attainments, setbacks and equity implications of Kenya’s social health insurance model
Source: BMJ Open. 2024 Dec 11;14(12):e085903. doi: 10.1136/bmjopen-2024-085903 (PMC11647346; doi:10.1136/bmjopen-2024-085903)
Supplement: Supplementary file 4 [file bmjopen-2024-085903.reviewer_comments.pdf]

PEER REVIEW HISTORY

BMJ Open publishes all reviews undertaken for accepted manuscripts. Reviewers are asked to complete a checklist review form and are provided with free text boxes to elaborate on their assessment. These free text comments are reproduced below.

ARTICLE DETAILS

Title (Provisional)

Social Health Insurance for Universal Health Coverage in LMICs; A retrospective policy analysis of attainments, setbacks, and equity implications of Kenya's Social Health Insurance Model.

Authors

Nungo, Susan; Filippon, Jonathan; Russo, Giuliano

VERSION 1 - REVIEW

**Reviewer**	**1**
**Name**	**Yazbeck, Abdo S.**
**Affiliation**	**Johns Hopkins University Bloomberg School of Public Health**
**Date**	**08-May-2024**
**COI**	**none**

This is an important paper about and important long term program (SHI in Kenya) that offers lessons to many countries considering a similar approach. If the authors can address two substantial flaws (relatively easy to fix) then the paper would be well worth publishing. I will focus here only on the two flaws and not on the many things I liked about the paper.

First, the opening sections somehow miss out on at least 4 critical papers on SHI in LMICs. As a result, the authors are far more positive about SHI as a health financing instrument for LMICs. The introduction section should be re-written to more accurately describe the limitations of SHI:

1. Savedoff, W.D., 2004. Is there a case for social insurance? Health Pol. Plann. 19 (3),

183–184. This is the definitive paper on the Latin American's long term experience with SHI and finds that SHI in LMICs produced both inequality and inefficiency

2. Wagstaff, A., 2010 May. Social Health Insurance Reexamined. Health Economics 19 (5), 503–517. Is the definitive summary of the European experience

3. Yazbeck, A.S., Savedoff, W.D., Hsiao, W.C., Kutzin, J., Soucat, A., Tandon, A., et al.,

2020. The case against labor-tax-financed social health insurance for low- and low middle-income countries. Health Aff. 39 (5), 892–897. May 1. Updated the earlier papers with more recent evidence. That paper also highlights the changes in SHI over the years that can make it less problematic to LMICs in some way

4. PMID: 36822716 "Addiction to a bad idea, especially in low- and middle-income countries: Contributory health insurance". Is a 2023 paper in SSM that narrows the attention to the link between contributions and access (a critical element of SHIs) and updates the empirical and theoretical challenges for SHI.

A second flaw with the paper is in the conclusion and is linked to the first flaw. The first sentence in the conclusion section is "SHI provides a sustainable path for financing healthcare in LMICs." That is an incorrect statement. As the paper itself argues, countries like Kenya with high informality in the labor market will struggle with SHI.

The policy focused qualitative approach is fairly informative about this important country experience, and if the two flaws are addressed adequately, will contribute to the field.

**Reviewer**	**2**
**Name**	**Park, Sewon**
**Affiliation**	**Ajou University School of Medicine, Medical Science**
**Date**	**27-Aug-2024**
**COI**	**No**

Major Revision

Methodology Response: P.6-10.

1. There is a lack of information on financial database analysis in ‘2.3 data collection’.

2. There is insufficient information on the final literature selection process for documental analysis (e.g. information on inclusion and exclusion criteria) and information about the analysis program is not clear.

Discussion Response: P.18-20.

1. How can future research address the methodological limitations of this study?

2. What are effective strategies to increase National Health Insurance Fund (NHIF) coverage among Kenya’s informal sector workers?

3. What are the specific measures to address inefficiencies arising in NHIF’s healthcare service purchasing and payment processes?

4. What implications does Kenya’s case have for social health insurance(SHI) implementation in other Low-and Middle-income Countries(LMICs)?

Minor Revision

Introduction and background Response: P.2-6.

1. A definition is needed for the acronym LMICs.

Methodology Response: P.6-10.

1. ‘Figure 1’ requires an indication of what is being referenced.

2. The numeric format error in ‘3.4 Ethics approval’ needs to be corrected.

VERSION 1 - AUTHOR RESPONSE

Reviewer 1:
Comments to the Author:
This is an important paper about and important long term program (SHI in Kenya) that offers lessons to many countries considering a similar approach. If the authors can address two substantial flaws (relatively easy to fix) then the paper would be well worth publishing. I will focus here only on the two flaws and not on the many things I liked about the paper.

First, the opening sections somehow miss out on at least 4 critical papers on SHI in LMICs. As a result, the authors are far more positive about SHI as a health financing instrument for LMICs. The introduction section should be re-written to more accurately describe the limitations of SHI:

1. Savedoff, W.D., 2004. Is there a case for social insurance? Health Pol. Plann. 19 (3), 183–184. This is the definitive paper on the Latin American's long term experience with SHI and finds that SHI in LMICs produced both inequality and inefficiency

2. Wagstaff, A., 2010 May. Social Health Insurance Reexamined. Health Economics 19 (5), 503–517. Is the definitive summary of the European experience

3. Yazbeck, A.S., Savedoff, W.D., Hsiao, W.C., Kutzin, J., Soucat, A., Tandon, A., et al., 2020. The case against labor-tax-financed social health insurance for low- and low middle-income countries. Health Aff. 39 (5), 892–897. May 1. Updated the earlier papers with more recent evidence. That paper also highlights the changes in SHI over the years that can make it less problematic to LMICs in some way

4. PMID: 36822716 "Addiction to a bad idea, especially in low- and middle-income countries: Contributory health insurance". Is a 2023 paper in SSM that narrows the attention to the link between contributions and access (a critical element of SHIs) and updates the empirical and theoretical challenges for SHI.

Response: Thank you for highlighting these very significant articles. The introduction has

now been rewritten to reflect the views presented in these four papers. The following lines and paragraphs have been added to the introduction section:

"…other evidence indicate a shift from payroll-based to general revenue-based mechanisms for financing health across countries in Europe where the SHI first started(1). This reverse direction by the Western world suggests a failure of the SHI to provide sustainable long-term health financing solutions. It therefore begs the question as to whether the SHI rush across LMICs is a misdirection that should be reassessed if LMICs are to make any progress toward universal coverage." (P.3, section 1 Introduction & background, paragraph 3)

"SHI can therefore widen the inequity gap and cause adverse impacts on the economy(2)." (P.4, section 1.1, paragraph 3)

"Additionally, Savedoff et al(3) rightly observe that cross-subsidization, as is intended in SHI, is not often adequately achieved due to the benefits packages attached to SHI. Health services provided by most benefit systems are normally foreseen and commonly demanded by the population and thus are not of insurable risk, leading to high premiums. Moreover, LMICs have fundamental limitations including inefficiencies in public health spending, a lack of political goodwill and poor governance which not only hinder any potential for a successful SHI, but can also increase health inequities and allow wastage of health resources(3)." (P.4, section 1.1, paragraph 4)

"However, other evidence suggest that by excluding populations that are most likely in need of healthcare services, SHI encourages inequity and hinders the UHC progress(2)." (P.4, section 1.1 paragraph 5)

"In their assessment of labor-based systems of financing healthcare, Yazbeck et al(4) argue that general revenues may be a more sustainable and efficient means of financing healthcare, than mechanisms that are based on employment status such as the SHI, and as such, LMICs aiming at achieving UHC should reassess their health financing choices. Additionally, reliance on labour-based systems provides an avenue for governments to defer the liability of paying for healthcare from the government to the few employed members of the population(3) such as in Thailand and Mexico where the ratio of social security spending per capita was at a point two and five times respectively, more than the government spending on health(3). Moreover, Wagstaff et al(1) highlight practices of evasion even within the formal sector using evidence from Vietnam, China, and the Philippines where formal sector enrolment was found to be just about 60-75%. This evidence suggests that the capacity to collect revenue through SHI systems is hugely limited, not only in the informal but within the formal sector as well." (P.4-5, section 1.1, paragraph 6).

Reviewer 1 comment 2:

A second flaw with the paper is in the conclusion and is linked to the first flaw. The first sentence in the conclusion section is "SHI provides a sustainable path for financing healthcare in LMICs." That is an incorrect statement. As the paper itself argues, countries like Kenya with high informality in the labor market will struggle with SHI.

The policy focused qualitative approach is fairly informative about this important country experience, and if the two flaws are addressed adequately, will contribute to the field.

Response:

Both perspectives have been presented better now, and the first paragraph of the conclusion has been rephrased as below:

"SHI can provide a path for financing healthcare and improving coverage in LMICs. Although significantly limited by its labor-based nature and other structural and contextual challenges, the risk minimization and resource pooling properties of the SHI allow for cross-subsidization of services, which if well executed, can improve healthcare access." (P.20, Conclusions, paragraph 1).

Reviewer: 2
Comments to the Author:
Major Revision
Methodology Response: P.6-10.
Reviewer 2 Comment 1. There is a lack of information on financial database analysis in '2.3 data collection'.

Response:

Thank you for the observant feedback. The terminology "financial database reviews" has been replaced with "review of financial reports and audited NHIF accounts" which are also mentioned among the documents that the search yielded for the document analysis in section 2.3.1. (P.8, section 2.3 Data collection, paragraph 1.)

Reviewer 2 Comment 2. There is insufficient information on the final literature selection process for documental analysis (e.g. information on inclusion and exclusion criteria) and information about the analysis program is not clear.

Response:

We agree that more information should be added about the inclusion and exclusion criteria for the literature selection process. This subsection has now been added under section 2.3.1 Document Analysis.

"Inclusion and Exclusion Criteria

The inclusion criteria for the document selection was that the articles had to be We modified the search to include only officially published reports from Kenya, official ministry guidelines, acts of parliament, or NHIF official publications. Unofficial reports and commentaries on Kenya's NHIF, blog posts, and social media posts were excluded from the final document analysis." (P.9, section 2.3.1 Document Analysis)

Section 2.4 Data Analysis has also been rephrased to improve clarity:

“Data from the document analysis and the interview transcription were fed into an Excel sheet and color-coded to represent either interview data or document review data. We employed the thematic approach to our data analysis. Themes and sub-themes were identified across both data sets. All data were analysed fairly and all common themes emerging from interviews with the different stakeholders were triangulated alongside those from document reviews…” (P.10, section 2.4 Data Analysis)

Discussion Response: P.18-20.

Reviewer 2 Comment 1. How can future research address the methodological limitations of this study?

Response:

Thank you for this comment, a sentence has now been added:

“Future research should expand the scope of stakeholders to obtain a more global perception of the SHI in Kenya.” (P.18, section 4 Discussion, paragraph 2)

Reviewer 2 Comment 2. What are effective strategies to increase National Health Insurance Fund (NHIF) coverage among Kenya’s informal sector workers?

Response:

This is an important point that is also addressed in section 4.1 Recommendations for Policy. We agree that it is important to include in the discussion as well and have now added the following sentences.

“Basing on the evidence from other countries that have made significant progress to UHC through co-financing their SHI with other government-funded options(5,6), co-financing mechanisms that cover the informal population, the poor and vulnerable residents should be improved to supplement the SHI.” (P.19, section 4 Discussion, paragraph 3)

Reviewer 2 Comment 3. What are the specific measures to address inefficiencies arising in NHIF’s healthcare service purchasing and payment processes?

Response:

Thank you for highlighting this information gap in this section. The following sentences have now been added to the discussion paragraph on inefficiencies in purchasing and payment.

“In addition to implementing SHI, health systems should implement strategic purchasing mechanisms and reinforce measures to ensure accountability and transparency to curb the inefficiencies, bureaucracies and misappropriation observed in the purchasing and payment system.” (P.19, section 4 Discussion, paragraph 4)

Reviewer 2 Comment 4. What implications does Kenya’s case have for social health insurance(SHI) implementation in other Low-and Middle-income Countries(LMICs)?

Response:

The following sentence has been added to the conclusion paragraph to address this important information gap on p.20 and the abstract conclusion on p.2.

"LMICs adopting SHI need to also implement co-financing arrangements, strategic purchasing systems, political goodwill, and good governance for the SHI systems to be beneficial." (P.2 & 20, Abstract conclusion and section 5 Conclusions)

Minor Revision. Introduction and background Response: P.2-6.
Reviewer 2 Comment 1. A definition is needed for the acronym LMICs.

Response:

Definition added: "Low and Middle-Income Countries, by the World Bank definitions, are countries that have a Gross National Income per capita of below $4,515 as of 2023(7)." P.3

Methodology Response: P.6-10.
Reviewer 2 Comment 1. 'Figure 1' requires an indication of what is being referenced.

Response: Figure 1 has now been referenced.

Reviewer 2 Comment 2. The numeric format error in '3.4 Ethics approval' needs to be corrected.

Response: The numeric format has been corrected to 2.5

References

1. Wagstaff A. Social health insurance reexamined. Health Econ. 2010;19(5):503–17.
2. Yazbeck AS, Soucat AL, Tandon A, Cashin C, Kutzin J, Watson J, et al. Addiction to a bad idea, especially in low- and middle-income countries: Contributory health insurance. Soc Sci Med 1982. 2023 Mar;320:115168.
3. Savedoff WD. Is there a case for social insurance? Health Policy Plan. 2004 May;19(3):183–4.
4. Yazbeck AS, Savedoff WD, Hsiao WC, Kutzin J, Soucat A, Tandon A, et al. The Case Against Labor-Tax-Financed Social Health Insurance For Low- And Low-Middle-Income Countries. Health Aff (Millwood). 2020 May;39(5):892–7.
5. Abiiro GA, McIntyre D. Achieving universal health care coverage: Current debates in Ghana on covering those outside the formal sector. BMC Int Health Hum Rights. 2012 Oct 29;12(1):25.
6. Gilardino RE, Valanzasca P, Rifkin SB. Has Latin America achieved universal health coverage yet? Lessons from four countries. Arch Public Health. 2022 Jan 21;80(1):38.

7. World Bank Country and Lending Groups – World Bank Data Help Desk [Internet]. [cited 2024 Oct 15]. Available from: https://datahelpdesk.worldbank.org/knowledgebase/articles/906519-world-bank-country-and-lending-groups
